# Supplementary material for: The causal role of circulating inflammatory markers in osteoporosis: a bidirectional Mendelian randomized study
Source: Front Immunol. 2024 Jul 18;15:1412298. doi: 10.3389/fimmu.2024.1412298 (PMC11291241; doi:10.3389/fimmu.2024.1412298)
Supplement: Supplementary file 1 [file DataSheet_1.zip › supplementary material/CIM-OSTEOPOROSIS /GCST90274831.csv_leave_one_out.pdf]

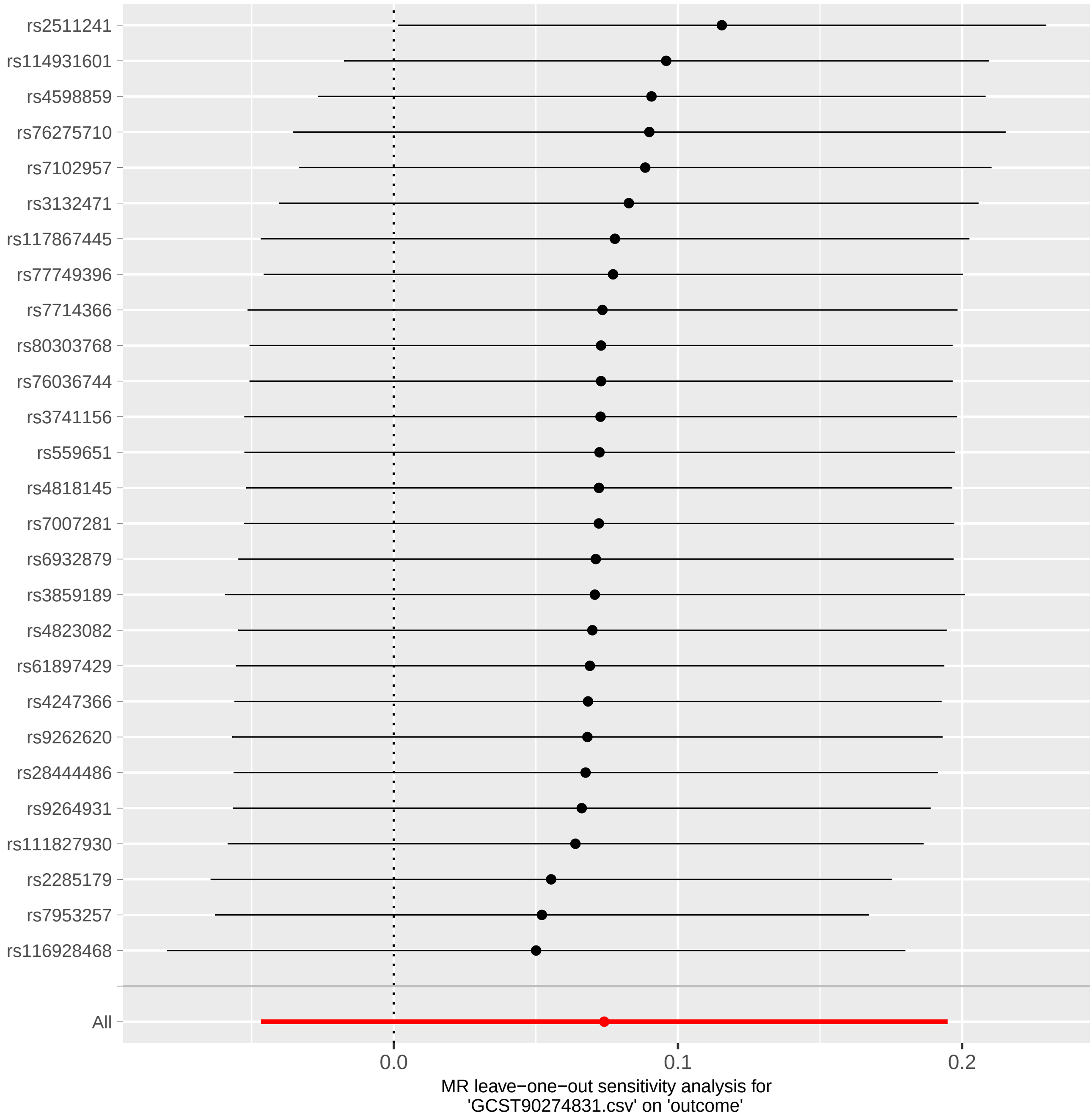
rs2511241
rs114931601
rs4598859
rs76275710
rs7102957
rs3132471
rs117867445
rs77749396
rs7714366
rs80303768
rs76036744
rs3741156
rs559651
rs4818145
rs7007281
rs6932879
rs3859189
rs4823082
rs61897429
rs4247366
rs9262620
rs28444486
rs9264931
rs111827930
rs2285179
rs7953257
rs116928468
All
0.0
0.1
0.2
MR leave−one−out sensitivity analysis for
'GCST90274831.csv' on 'outcome'
